# Countermovement push-up test to assess the upper extremity force-time characteristics in swimmers during a macrocycle

**DOI:** 10.1371/journal.pone.0289573

**Published:** 2023-08-03

**Authors:** Ferhat Öztürk, Evrim Ünver, Aykut Özçadırcı, Şükrü Alpan Cinemre, Gizem İrem Kınıklı

**Affiliations:** 1 Faculty of Physical Therapy and Rehabilitation, Hacettepe University, Ankara, Türkiye; 2 Faculty of Sport Sciences, Hacettepe University, Ankara, Türkiye; University of West Attica, GREECE

## Abstract

Although it is known that swimming training can improve upper extremity performance, the force-time characteristics of the upper extremity during different training periods are not well understood. The objective of this study was to measure changes in the force-time characteristics of the upper extremity of young swimmers during different training periods within a season. Seventeen young swimmers, comprising 5 males (age: 15.4 ± 0.54 years); 12 females (16.4 ± 2.6 years) participated in this study. They were tested at four experimental test time points: baseline (E1), post-general preparation (E2), post-specific preparation (E3), and taper season (E4). The countermovement push-up test was performed using a force plate to measure force time parameters. Differences in force, time, velocity and impulse parameters were evaluated between the different periods. The study found that vertical take off velocity significantly increased across the assessed periods (F = 11.79; p = .001; η^2^ = .424), with significant increases from E1 to E2 (p < .001) and from E3 to E4 (p = .016). Flight Time also significantly increased across the assessed periods (F = 11.79; p = .001; η^2^ = .424), with significant increases from E1 to E2 (p < .001), from E1 to E4(p = .001), and from E3 to E4 (p = .005). The Force Impulse significantly increased throughout the assessed periods (F = 5.84; p = .012; η^2^ = .267), with significant increases from E1 to E2, (p = .006), from E1 to E3 (p = .016), and from E1 to E4 (p = .003). As this study shows, periods of increased training intensity can affect athletic progression, even though training aims to improve strength, speed, and performance. While some practical aspects such as strength, flight time, and impulse parameters may change during a macrocycle, the countermovement push-up test can provide trainers with an alternative and convenient way to monitor anaerobic force, speed, and performance, as well as measure explosive force-time performance in the upper body.

## Introduction

Swimming is a cyclical sport that involves constant movement of the extremities, making performance dependent on various factors. However, each of the traditional propulsion techniques presents biomechanical variability and exhibits similar but not identical sequential cycles [1,2]. In this sport, where water provides significantly more resistance to athletes’ movements, requiring them to constantly generate propulsion [3]. Because of these repeated repetitive movements, upper extremity strength and force are critical for success in swimming.

The upper extremity force-time characteristics is considered an essential parameter of athletic performance in swimming [4]. Evaluating an athlete’s force-time parameters is crucial to determine their performance and monitoring injury prevention strategies and training programs’ short- and long-term effects [5]. Since the force parameters, particularly those generated during explosive movements, are related to joint stability, the ability to produce force rapidly (within 25–50 ms) is more important than maximal isometric voluntary force capabilities produced through isometric or isokinetic exercises for injury prevention [5,6]. Gradual improvement in upper extremity force-time and power outputs can occur in response to appropriate training stress [7,8]. However, optimal power assessment can be complex. In the past, medicine ball throws, bench presses, and the number of push-ups performed in a given periodic time was used to monitor and evaluate the effectiveness of training on upper extremity muscle performance [9–11]. More recently, force platforms have been used to evaluate upper extremity force-time and power outputs through countermovement and plyometric push-up tests [12–14].

In competitive swimming, coaches and athletes often organize their training programs periodically to maximize performance, from beginners to elite competitions. Programs usually increase the workload (training distance and intensity) for most of the season and then reduce the load at the end of the season. The periodization system is based on macrocycles, which usually consist of three training periods per year, depending on the annual calendar of the main competitions [15]. Trainers divide a macrocycle into general preparation, specific preparation, and taper periods by changing the volume, intensity and/or training frequency. Changes in upper extremity strength and power parameters affect swimming performance as force and strength are significantly correlated with swimming performance [3]. Therefore, it is crucial that coaches understand this relationship and incorporate periodically scheduled training in a training macrocycle. Additionally, performance-based changes may occur not only due to insufficient training but also due to different training loads (intensity and volume) applied in different periods of a given macrocycle [16].

During a macrocycle, different training contexts can highlight the exploration of various physiological, strength, power, speed, and performance adaptations, which require further study to deepen the knowledge gained in this process [17]. Further research is needed to develop more efficient and adequate training methods for young swimmers, whose characteristics are currently understudied [18–20]. The evaluation and design of swimming training programs should consider swimmers’ physical condition and force-time curve characteristics. These force-time curve parameters are essential capacities of the neuromuscular system to develop maximal strength rapidly and are associated with athletic performance in swimmers [21,22]. However, no previous research has investigated the effect of training periods (periodization) on the force-time parameters in swimmers. Such information can be crucial to understanding the effect of training, risk factors for injury, and monitoring, individually and as a team. Therefore, this study aimed to measure the changes in the upper extremity performance parameters in the force-time profile of young swimmers at different training periods (general preparation, specific preparation, and taper) of the first macrocycle of the season. We hypothesized that there would be differences in the upper extremity force-time profile at different training periods during a macrocycle in swimmers and that the force-time parameters would increase at the end of the macrocycle.

## Materials and methods

### Experimental approach to the problem

This study was designed as a single-group prospective cohort, repeated measures in young swimmers. All eligible swimmers and their legal guardians were informed of the study procedures and provided written consent. The study was approved by the Hacettepe University Non-Invasive Clinical Research Ethics Committee **(**approval number: GO 21/1151) and was conducted in accordance with the Declaration of Helsinki. The study was also registered at clinicaltrials.gov (NCT05691673). Swimmers were assessed in the traditional 3-term preparation program (1 competition season, 16 weeks) at the beginning of the season and after the general preparation, specific preparation, and the Taper periods. The swimmers were tested at each experimental test time point (Pre-season: E1, post-general preparation: E2, post-specific: E3, and post-Taper season: E4), at the same time of day, on the first training day of the week and after two days of rest. During each test, swimmers performed three countermovement push-up attempts. In E1, 24 swimmers were tested. Two swimmers did not participate in the E2, one in E3, and four in the E4. Swimmers who missed their test sessions were excluded from the study.

During the 16-week training season, the swimmers completed all swim training twice a day on Tuesday, Thursday, and Saturday and once a day on Wednesday, Friday, and Sunday. They also completed dry land training once a day on Wednesday, Friday, and Sunday as designated by the coach**.** Throughout the macrocycle, all swimmers completed coach-designated land-based training that included general strengthening and stretching exercises for the trunk and upper/lower limbs, such as core, squats, pull-ups, cleans, and chin-ups**.** The coach also briefed the swimmers on the importance of sleep, stress, and nutrition. The training season is divided into several periods in line with specific goals. The general preparation period is 1–6 weeks and includes light to moderate training volume and intensity to improve aerobic capacity and strength. The specific preparation period takes place during weeks 7–12 and includes intensive training volume and intensity to improve strength and anaerobic-aerobic conditions. The taper period during weeks 13–16 focuses on achieving maximum performance with low training volume. This period optimizes the acquired physical and technical conditions by removing the fatigue caused by previous intense training loads.

The adolescent team athletes attended the training sessions without interruption. Each swimmer performed warm-up and countermovement push-up trials one week prior to the pre-season test to familiarize themselves with the equipment and procedures. In all measurements, three repetitive trials were taken after a 15-minute warm-up. Participants were asked to avoid severe physical activity within 24 hours prior to the tests, given guidance on hydration, and verbally encouraged during the tests. Measurements were taken on Tuesdays after 48 hours of rest (Sunday-Tuesday) at the start of each period to eliminate acute fatigue.

### Participants

Seventeen young swimmers participated in this study, consisting of 5 males (age: 15.4 ± 0.84 years, body mass index: 21.24 kg/m2, height: 175.5 ± 10.7 cm) and 12 females (age:15.7 ± 0.96 years, body mass index: 21.49 kg/m2, height: 169.4 ± 5.36 cm). Their best performance at the short course (25 m) for the 50-m freestyle was 28.45 ± 1.31 sec at the beginning of the season (355 points for males and 523 points for females at the start of the season FINA (Fédération Internationale de Natation, 2021) point score) and 27.33 ± 0.98 sec at the end of the season (401 points for male and 590 points for female at the beginning of the season at FINA point), respectively. Although the swimmers in the team entered the same training sessions, each swimmer had an individual branch represented. Eleven swimmers were freestyle, two were breaststroke, and four were backstroke and butterfly style. The swimmers participating in the study had at least five years of competitive swimming experience. They had done 7–9 swimming sessions and 2–3 hours of dry field training per week under the supervision of the same trainer. The study did not include swimmers who had undergone upper extremity surgery within the last year and those who experienced pain during the tests.

### Procedure

**Fig 1:**
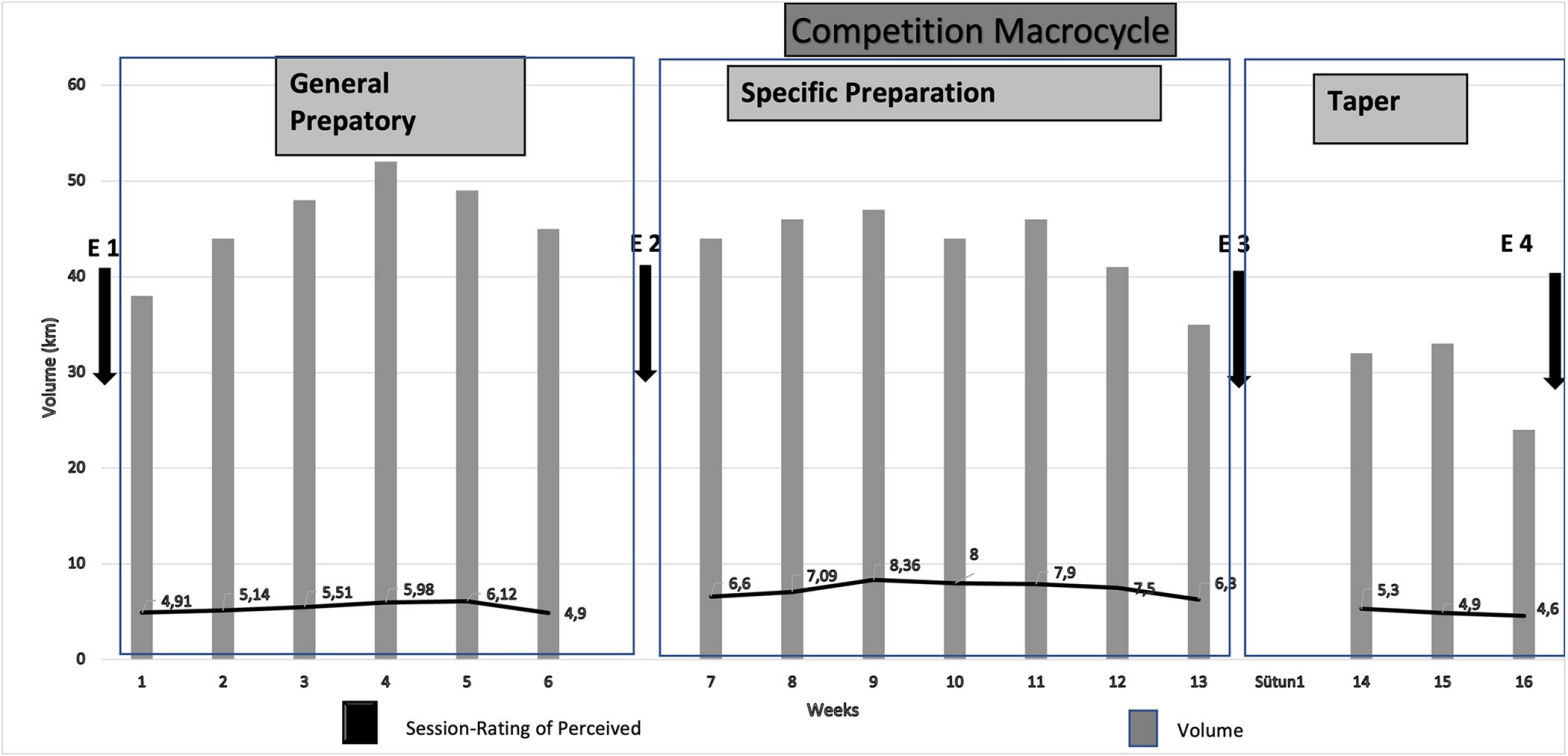
Weekly volume and Sessions—Rating of Perceived Exertion during the first macrocycle (16 weeks) of the training season.

The swimming coach recorded the distance swam in kilometers after each training session each day for seven days to determine the weekly training volume. The total distance for the week was calculated as the weekly volume. Fig 1 shows the training volume over the 16-week macrocycle. Height, body mass, and body mass index were measured at each assessment by a 6-year physical therapist.

The kinetic parameters of the countermovement push-up test were measured using a force platform (Kistler type 9260AA; Kistler, Switzerland) and Bioware Software (version 5.11; Bioware Software, Switzerland) at a frequency of 2000 Hz. Before each test, participants performed 15 minutes of warm-up exercises under the supervision of a coach.

**Fig 2:**
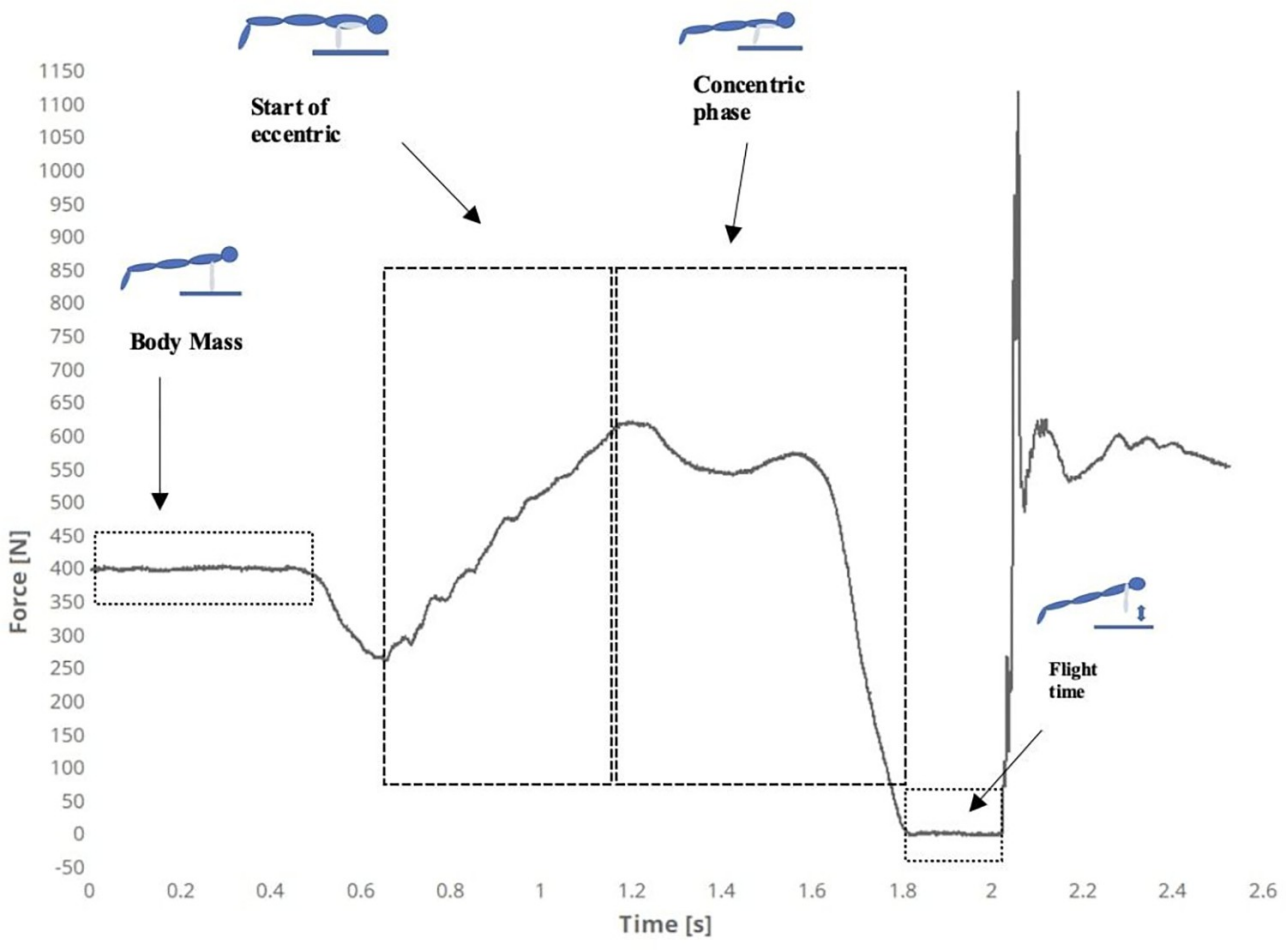
Example of force-time curve graphic obtained from countermovement push up.

These exercises included circumduction movements, upper extremity flexion-extension, dynamic stretching (trapezius, pectoral muscles, elbow and wrist flexors, and extensors), and countermovement push-ups at a submaximal level. After a 5-minute rest break, participants performed three maximal countermovement push-up tests with a 60-second rest break between repetitions. They were instructed to perform the test quickly and maximally and to push themselves as high as possible with their hands at their chosen width. Participants were positioned in 90 degrees of shoulder flexion, elbow, trunk, and hip in full extension, feet together on the ground, and body weight on toes (Fig 2). After a 3-second count, the participants quickly brought their trunks closer to the platform and pushed themselves vertically upwards. No hints were given for landing, and they were only asked to fall on the platform with both hands, return to the starting position after landing, and wait for 2 seconds. The data were filtered using a Butterworth filter with a cutoff frequency of 8 Hz. The kinetics data obtained included relative and absolute maximal force, rate of force development (RFD), force impulse (FI), vertical takeoff velocity**,** average force, and average velocity. The kinematics data included flight time (FT) and were taken from the force-time curve. The two best attempts from the highest values of FT were averaged for subsequent analysis.

Before beginning the current study, 35 swimmers underwent two countermovement push-up tests seven days apart to assess test-retest reliability. High reliability was found for force and impulse parameters **(**Flight Time (s): ICC: 0.914, CV%: 5.6, Mean Force (N): ICC = 0.99, CV% = 9, Peak Force (N): ICC: 0.972, CV%: 8.5, Rate of Force Development (N/s): ICC: 0.717, CV%: 10.4, Impulse (N.s): ICC: 0.897, CV% = 6.0).

### Statistical analyses

The statistical analyses were conducted using SPSS 23.0 software (IBM SPSS Statistics version 23.0, IBM Corp. Armonk, New York, USA) s. The normal distribution of variables was assessed using Shapiro-Wilks analytical methods. Differences between periods (baseline (E1), after general preparation (E2), after specific preparation (E3), and after the taper period (E4) were analyzed using repeated-measures analysis of variance and Bonferroni post hoc tests. The eta-squared (η^2^) test was used to determine the percentage of the variance explained by each covariate (effect size).The following interpretations were used: 0< η^2^ <0.04 (trivial effect), 0.04<η^2^ ≤0.24 (small effect), 0.25≤η^2^ < 0.64 (moderate effect), and η^2^ > 0.64 (large effect) [23].

## Results

The coaches determined the average weekly volume (km) for each training period to be 46.22 ± 4.85 km (preparation), 44.16 ± 2.66 km (specific), and 29 ± 6.21 km (taper). The volume was the same for all swimmers.

The comparison of force-time parameters along the training periods of the macrocycle is presented in Table 1.

**Table 1:** Data variation (repeated-measures ANOVA) and changes in force-time parameters obtained from force platform by swimmers over the first macrocycle of the training season.

Variable	Time Effect				Baseline (E1)		Preparatory (E2)		Specific (E3)		Taper (E4)	
	F	p	η^2^	Power	Mean	SD (±)	Mean	SD (±)	Mean	SD (±)	Mean	SD (±)
**Force Parameters**												
**Relative max Force (%BW)**	1.04	.383	.061	.27	157.95	16.2	160.24	15.9	155.2	20.3	155.50	12.2
**Peak Force (N)**	.064	.855	.004	.057	638.25	126.41	637.98	119.05	640.68	161.70	631.32	104.54
**Vertical Take Off Velocity (m/s)**	11.79	.001*	.424	.989	.87	.33	1.16¤	.38	1.03¤	.20	1.12¤†	.25
**Average Force (N)**	1.53	.234	.088	.266	480.8	100	474.9	85.9	498.7	89.9	489.5	78.6
**Average velocity (m/s)**	2.00	.126	.111	.483	1.18	.40	1.20	.57	1.10	.43	1.32	.43
**Max. Rate of Force Development (N/s)**	.418	.741	.025	.127	2841	963	2875	912	2915	1116	2628	703
**Time Parameter**												
**Flight time (s)**	11.79	.001*	.424	.970	.183	.06	.230¤	.07	.21	.042	.23¤†	.05
**Force Impulse Parameter**												
**Force Impulse from Flight T [Ns]**	5.84	.012*	.267	.777	37.71	16.2	47.04¤	21.9	44.89¤	11.6	47.02¤	12.9

SD: Standard deviation.

*Significant time effect (p<0.05).

¤Significant difference between E1—E2, E1—E3, E1—E4 (p<0.05).

§Significant difference between E2—E3 or E2—E4 (p<0.05).

†Significant difference between E3—E4 (p<0.05).

Relative max force, peak force, average force, and average velocity were similar in all periods (p> .05). The mean Vertical take-off velocity increased 33.3% (p< .001) from E1 to the E2 and 8.7% (p = .016) from E3 to E4. The mean Flight Time increased 25.6% (p < .001) from E1 to E2, 25% (p = .001) from E1 to E4, and 9.5% (p = .005) from E3 to E4. The mean Force Impulse increased 24.7% (p = .006) from E1 to the E2, 19.04% (p = .016) from E1 to E3, and 24.6% (p = .003) from E1 to E4.

## Discussion

This study aimed to measure differences in upper extremity performance parameters, specifically force-time characteristics among young swimmers at different training periods in the first macrocycle of the season. The current study found that a well-designed year-long plan significantly improved these force-time parameters. Furthermore, the study demonstrates that intensity and high-volume training performed during the specific preparation period affect the improvements in these force-time parameters. We anticipated variations in force-time parameters of the upper extremities due to the different training targets during each training period of swimmers. These findings partially support our hypothesis that force-time parameters differ between training periods (baseline, preparatory, specific preparatory, and taper periods).

Competitive swimming aims to maximize the swimmer’s thrust by leveraging technique and energy capacity to finish the race as quickly as possible [24]. Therefore, using an assessment that measures force and propulsion makes sense. In the current study, average changes in swimmers of specific age groups were observed during a training macrocycle, which may be beneficial for coaches. However, it is crucial to consider that participants’ competitiveness and other factors may contribute to these changes [18]. Additionally, it should be noted that training programs are similar in intensity and volume for all swimmers.

The increase in certain force variables and the corresponding decrease in 50-m swimming time throughout the season suggest that trainers should prioritize strength development in addition to the technical development of swimmers in this age group. It is important to note that, in addition to improvements in swimming performance, physiological variables likely play a crucial role in optimizing short- and medium-distance swimming performance. To achieve optimal performance development, coaches can begin with technical training at the start of the season and gradually increase training intensity over time.

In recent years, countermovement push-ups performed on a force plate have become increasingly popular for monitoring upper extremity power in athletes [13,14,25,26]. Suchomel et al. conducted a study to evaluate the reliability of various push-up exercises, including static, countermovement, and drop push-ups, in terms of measuring variables such as peak force (PF), rate of force development (RFD), and peak power (PP) [26]. The study focused on a group of well-trained male gymnasts with an average age of 15.1 ± 1.7 years. The findings indicated that all three techniques—static, countermovement, and drop push-ups—demonstrated reliable results in assessing PF, RFD, and PP among young gymnasts. Numerous additional studies have investigated various iterations of the Plyometric Push-Up (PPU) test in adult populations. These studies have aimed to explore different versions or adaptations of the PPU test and their impact on performance outcomes. Hogarth et al. and Hrysomallis and Kidgell independently reported that the Plyometric Push-Up (PPU) test demonstrated reliability in assessing variables such as peak force (PF) and peak rate of force development (pRFD) [13,27]. These studies provided evidence supporting the reliability of the PPU test in determining these parameters. Wang et al. corroborated these findings and expanded upon them by demonstrating that variables such as average power (AP) and peak power (PP) were also reliable measures during the PPU test in adult populations [28]. Parry et al. asserted that force-time parameters, including flight time, peak force, mean force, and impulse, obtained from force plates, are reliable indicators for evaluating upper body performance in athletes [12]. The study highlighted the dependability of these parameters in assessing and quantifying the performance capabilities of the upper body. This study is the first to examine the upper extremities’ force-time parameters using the countermovement push-up performed on a force plate, specifically in swimmers. While countermovement push-ups conducted on force plates have gained popularity for evaluating upper extremity performance, the current study represents a novel contribution by focusing on swimmers and exploring the force-time characteristics of their upper extremities during this exercise.

Due to the limited number of studies on periodization in young swimmers, limited guidelines are available on training periodization. Additionally, during adolescence, maturation progression is known to affect performance, physiological variables (such as cardiovascular adaptations) [29,30] and biomechanical variables (such as stroke index, stroke length and stroke rate) [31,32]. Studies in the literature have generally associated biomechanical, physiological, and anthropometric changes in the performance of young swimmers with swimming performance cross-sectionally [32–34] and longitudinally during a macrocycle [20,29,35,36]. This current study is the first to examine the force-time parameters during a macrocycle.

The general preparation phase aims to prepare athletes for the next period of intense training physically. Endurance, speed, strength, and flexibility are among the goals [15]. Although high-intensity strength training is not the main focus during the general preparation period, the increase in strength parameters observed after this period in the current study may be due to a transition period at the beginning of the macrocycle. This phenomenon could explain the weak performances at the beginning of the season reported in the literature [37,38].

During the specific preparation period, athletes should focus on developing their core endurance and their aerobic and anaerobic muscular endurance, muscle strength, and speed. This period involves increased training volume and intensity, resulting in a heavy workload. It is common for swimmers to generate less power during the heaviest training phase [15]. Our study found no increase in force, time, and impulse parameters, despite the intense training during the specific preparation period, which may be attributed to fatigue. Increases in the intensity and volume of periodic strength programs are likely to cause acute and chronic fatigue in swimmers, which can over-stress the neuroendocrine system [15]. Improvement in high-volume workload-based swim training programs is often expected after the taper period. According to Thomas et al. [39]**,** an adequate taper period (21–28 days) with a reduced training load is required for swimming performance to benefit from overloading. Our study shows an increase in strength parameters between the E3-E4 periods, which indicates a taper period, which is characterized by low volume and low training load in the macrocycle [37,40]. Tucher et al. [20] found that blood lactate concentration decreased after the competition period compared to the general preparation period. This result supports our study, showing that low-volume training during the specific preparation period until after the taper can reduce fatigue. Zacca et al. found that energetics and anthropometric values were higher during the specific preparation and post-competition periods compared to the general preparation period in 3-term, 16-week, and 48-week macrocycles in young swimmers [29,41]. Batalha et al. reported that swimming through a macrocycle strengthened internal rotators in one season [42]. Considering the effect of internal rotators in the counter movement push-up test, this strength development may have affected the results of the current study. Furthermore, decreased strength values after the specific preparation period are compatible with a heavy workload. Swimmers are expected to generate less power during the heaviest training phase.

This study has several limitations. Firstly, it is related to the individual characteristics of the swimmer group participating in the research. Therefore, caution should be exercised when applying these findings to swimmers of varying competitiveness. Secondly, this study did not evaluate other performance measures, such as stroke rate or length, nor did it provide biomechanical swimming evaluations. It would be beneficial to collect this data to test the findings’ relevance to swimmers’ performance. Furthermore, while this study reported statistically significant findings, they may not be considered "practically significant." Therefore, it is essential to interpret the results correctly when applying them to practice. Another limitation was that the 50m performance was only evaluated at the beginning and end of the season. Since performance improvement is expected in a training season, evaluating how training periods affect performance would be better. As training is a dynamic process, the data obtained from the strength platform in this study can provide reliable insights into swimmer development. It can also help determine the factors that impact basic swimming performance and their relationship with the training program. Future studies should examine parameters related to the force-time profile in seasonal macrocycles across different age groups and genders.

## Conclusions

Throughout the season, swimmers showed an increase in force, time, and impulse parameters from countermovement push-up (CMPU) during the first macrocycle. Specifically, vertical take-off velocity, flight time, and force impulse parameters increased throughout the season, except before and after the specific preparation period. This study highlights that practical aspects such as vertical take-off velocity, flight time, and impulse parameters can change over a macrocycle. As a result, trainers may use the countermovement push-up test as an alternative and useful tool to monitor force, time, velocity, and performance throughout the training process. However, since the countermovement push-up test requires maximum performance, trainers should consider the participant’s motivation and ability to perform at their maximum during the test. Therefore, tests should be incorporated into the seasonal training plan and carefully planned within macro-cycle periods to avoid misinterpretation of data. Additionally, fatigue resulting from increased volume and intensity during the specific preparation period in a macrocycle affected the force parameters.

## Supporting information

S1 Dataset (TIFF)

## References

[1] Preatoni E., Hamill J., Harrison A. J., Hayes K., Van Emmerik R. E., Wilson C. et al. Movement variability and skills monitoring in sports. Sports Biomech. 2013;12(2):69–92. doi: 10.1080/14763141.2012.738700 23898682

[2] Fernandes A., Mezêncio B., Soares S., Duarte Carvalho D., Silva A., Vilas-Boas et al. Intra-and inter-cycle velocity variations in sprint front crawl swimming. Sports Biomech. 2022, 1–14. 10.1080/14763141.2022.2077815 35659480

[3] Smith D.J., Norris S.R., and Hogg J.M., Performance evaluation of swimmers. Sports Med., 2002. 32(9): 539–554. 12096928 10.2165/00007256-200232090-00001

[4] Thng S., Pearson S., Rathbone E., & Keogh J. W., The prediction of swim start performance based on squat jump force-time characteristics. PeerJ, 2020. 8: e9208. doi: 10.7717/peerj.9208 32547864 PMC7271885

[5] Buckthorpe M. and Roi G.S., The time has come to incorporate a greater focus on rate of force development training in the sports injury rehabilitation process. M.L.T.J. 2017. 7(3): 435. doi: 10.11138/mltj/2017.7.3.435 29387636 PMC5774916

[6] Solomonow M. and Krogsgaard M., Sensorimotor control of knee stability. A review. Scand J Med Sci Sports: Review Article, 2001. 11(2): 64–80. doi: 10.1034/j.1600-0838.2001.011002064.x 11252464

[7] Dobbs I. J., Oliver J. L., Wong M. A., Moore I. S., & Lloyd R. S., Effects of a 12-week training program on isometric and dynamic force-time characteristics in pre–and post–peak height velocity male athletes. JSCR, 2020. 34(3): 653–662. doi: 10.1519/JSC.0000000000003467 31904716

[8] Jones J. V., Pyne D. B., Haff G. G., & Newton R. U., Comparison between elite and subelite swimmers on dry land and tumble turn leg extensor force-time characteristics. JSCR, 2018. 32(6): 1762–1769. doi: 10.1519/JSC.0000000000002041 29786631

[9] Gonjo T., Eriksrud O., Papoutsis F., & Olstad B. H., Relationships between a load-velocity profile and sprint performance in butterfly swimming. Int J. Sports Med, 2020. 41(07): 461–467. doi: 10.1055/a-1103-2114 32059244

[10] Stockbrugger B.A. and Haennel R.G., Validity and reliability of a medicine ball explosive power test.JSCR, 2001. 15(4): 431–438. 11726253

[11] McGuigan M.R., Wright G.A., and Fleck S.J., Strength training for athletes: does it really help sports performance? Int J Sports Physiol Perform, 2012. 7(1): 2–5. doi: 10.1123/ijspp.7.1.2 22461461

[12] Parry G.N., Herrington L.C., and Horsley I.G., The test–retest reliability of force plate–derived parameters of the countermovement push-up as a power assessment tool. J. Sport Rehabil., 2020. 29(3): 381–383. doi: 10.1123/jsr.2018-0419 31628273

[13] Hogarth L., Deakin G., and Sinclair W., Are plyometric push-ups a reliable power assessment tool? JASC, 2013. 21: 67–69.

[14] Koch J., Riemann B.L., and Davies G.J., Ground reaction force patterns in plyometric push-ups. JSCR, 2012. 26(8): 2220–2227. doi: 10.1519/JSC.0b013e318239f867 21986698

[15] Turner A., The science and practice of periodization: a brief review. Strength Cond J, 2011. 33(1): 34–46.

[16] B Barbosa T. M., Morais J. E., Marques M. C., Silva A. J., Marinho D. A., & Kee Y. H., et al., Hydrodynamic profile of young swimmers: Changes over a competitive season. Scand J Med Sci Sports, 2015. 25(2): e184–e196. doi: 10.1111/sms.12281 24975756

[17] Costa M. J., Bragada J. A., Mejias J. E., Louro H., Marinho D. A., Silva A. J. et al., Effects of swim training on energetics and performance. Int J of Sports Med, 2013. 34(06): 507–513. doi: 10.1055/s-0032-1327573 23180214

[18] Morais J. E., Marques M. C., Marinho D. A., Silva A. J., & Barbosa T. M,Longitudinal modeling in sports: Young swimmers’ performance and biomechanics profile. Hum. Mov. Sci., 2014. 37: 111–122. doi: 10.1016/j.humov.2014.07.005 25150801

[19] Lätt E., Jürimäe J., Mäestu J., Purge P., Rämson R., Haljaste. et al., Physiological, biomechanical and anthropometrical predictors of sprint swimming performance in adolescent swimmers. J Sports Sci Med, 2010. 9(3): 398. 24149633 PMC3761703

[20] Tucher G., Castro F. A. D. S., Garrido N., & Fernandes R., Monitoring Changes Over a Training Macrocycle in Regional Age‐Group Swimmers. J. Hum. Kinet., 2019. 69(1): 213–223. 31666903 10.2478/hukin-2019-0014 PMC6815095

[21] Papadopoulos C., Sambanis M., Gissis I., Noussios G., Gandiraga E., Manolopoulos, et al., Evaluation of force and vertical jump performance in young swimmers with different force-time curve characteristics. Biol. Sport, 2009. 26(4): 301.

[22] Ling B. H., Blanksby B., Elliott B., & McElroy G. K., Force-time characteristics of the butterfly turns by age-group swimmers. J. Hum. Mov. Stud., 2004. 47(5): 429–451.

[23] An effect size primer: A guide for clinicians and researchers Professional Psychology: Research and Practice, 40 (2009), pp. 532–538.

[24] Figueiredo P., Toussaint H. M., Vilas-Boas J. P., & Fernandes R. J., Relation between efficiency and energy cost with coordination in aquatic locomotion. Eur. J. Appl. Physiol, 2013. 113(3): 651–659. 10.1007/s00421-012-2468-8 22903863

[25] Gillen Z. M., Miramonti A. A., McKay B. D., Jenkins N. D., Leutzinger T. J., & Cramer J. T., Reliability and sensitivity of the power push-up test for upper-body strength and power in 6–15-year-old male athletes. JSCR, 2018. 32(1): 83–96. doi: 10.1519/JSC.0000000000002313 29084096

[26] Suchomel T.J., Sands W.A., and McNeal J.R., Comparison Of Static, Countermovement, And Drop Jumps Of The Upper And Lower Extremities In Us Junior National Team Male Gymnasts. Sci. Gymnast. J., 2016. 8(1):15–30

[27] Hrysomallis C. and Kidgell D., Effect of heavy dynamic resistive exercise on acute upper-body power. JSCR, 2001. 15(4): 426–430. 11726252

[28] Wang R., Hoffman J. R., Sadres E., Bartolomei S., Muddle T. W., Fukuda et al., Evaluating upper-body strength and power from a single test: The ballistic push-up. JSCR, 2017. 31(5): 1338–1345. doi: 10.1519/JSC.0000000000001832 28166187

[29] Zacca R., Azevedo R., Ramos V. R. Jr, Abraldes J. A., Vilas-Boas J. P., de Souza Castro, et al., Biophysical follow-up of age-group swimmers during a traditional three-peak preparation program. JSCR, 2020. 34(9): 2585–2595. doi: 10.1519/JSC.0000000000002964 30640304

[30] Obert P., Mandigout S., Vinet A., N’guyen L. D., Stecken F., & Courteix., Effect of aerobic training and detraining on left ventricular dimensions and diastolic function in prepubertal boys and girls. Int. j Sports Med., 2001. 22(02): 90–96. doi: 10.1055/s-2001-11343 11281623

[31] Lätt E., Jürimäe J., Haljaste K., Cicchella A., Purge P., & Jürimäe T, Longitudinal development of physical and performance parameters during biological maturation of young male swimmers. Percept. Mot Skills, 2009. 108(1): 297–307. doi: 10.2466/PMS.108.1.297-307 19425470

[32] Mezzaroba P.V. and Machado F.A., Effect of age, anthropometry, and distance in stroke parameters of young swimmers. Int J Sports Physiol Perform, 2014. 9(4): 702–706. doi: 10.1123/ijspp.2013-0278 24231272

[33] Figueiredo P., Silva A., Sampaio A., Vilas-Boas J. P., & Fernandes R. J., Front crawl sprint performance: A cluster analysis of biomechanics, energetics, coordinative, and anthropometric determinants in young swimmers. MC, 2016. 20(3): 209–221. doi: 10.1123/mc.2014-0050 26061270

[34] Morais J. E., Saavedra J. M., Costa M. J., Silva A. J., Marinho D. A., & Barbosa T. M., Tracking young talented swimmers: follow-up of performance and its biomechanical determinant factors. Acta Bioeng. Biomech., 2013. 15(3): 129–138. 24215298

[35] Ferreira S., Carvalho D., Monteiro A. S., Abraldes J. A., Vilas-Boas J. P., Toubekis, et al., Physiological and biomechanical evaluation of a training macrocycle in children swimmers. Sports, 2019. 7(3): 57. doi: 10.3390/sports7030057 30836622 PMC6473474

[36] Ferreira S., Carvalho D. D., Cardoso R., Rios M., Soares S., Toubekis, et al., Young swimmers’ middle-distance performance variation within a training season. Int. J. Environ. Res. Public Health, 2021. 18(3): 1010. doi: 10.3390/ijerph18031010 33498817 PMC7908489

[37] Anderson M. E., Hopkins W. G., Roberts A. D., & Pyne D. B., Monitoring seasonal and long-term changes in test performance in elite swimmers. Eur J Sport Sci, 2006. 6(3): 145–154.

[38] Mujika I. and Padilla S., Detraining: loss of training-induced physiological and performance adaptations. Part I. Sports Med., 2000. 30(2): 79–87. 10966148 10.2165/00007256-200030020-00002

[39] Thomas L., Mujika I., and Busso T., A model study of optimal training reduction during pre-event taper in elite swimmers. J. Sports Sci, 2008. 26(6): 643–652. doi: 10.1080/02640410701716782 18344135

[40] Bonifazi M., Sardella F., and Lupo C., Preparatory versus main competitions: differences in performances, lactate responses and pre-competition plasma cortisol concentrations in elite male swimmers. Eur. J. Appl. Physiol., 2000. 82(5): 368–373. 10.1007/s004210000230 10985589

[41] Zacca R., Azevedo R., Chainok P., Vilas-Boas J. P., Castro F. A. D. S., Monitoring age-group swimmers over a training macrocycle: energetics, technique, and anthropometrics. JSCR, 2020. 34(3): 818–827. 10.1519/JSC.0000000000002762 30113917

[42] Batalha N., Marmeleira J., Garrido N., & Silva A. J., Does a water-training macrocycle really create imbalances in swimmers’ shoulder rotator muscles? Eur J Sport Sci., 2015. 15(2): 167–172. doi: 10.1080/17461391.2014.908957 24754705

