# Correction: Countermovement push-up test to assess the upper extremity force-time characteristics in swimmers during a macrocycle

**DOI:** 10.1371/journal.pone.0310032

**Published:** 2024-09-05

**Authors:** Ferhat Öztürk, Evrim Ünver, Aykut Özçadırcı, Şükrü Alpan Cinemre, Gizem İrem Kınıklı

There are errors in the Funding statement. The correct Funding statement is as follows: This study was partially supported by the TÜBİTAK within the scope of project no 219S813. No additional external funding was received for this study.
