# Supplementary figures and images for: Countermovement push-up test to assess the upper extremity force-time characteristics in swimmers during a macrocycle
Source: PLoS One. 2023 Aug 3;18(8):e0289573. doi: 10.1371/journal.pone.0289573 (PMC10399821; doi:10.1371/journal.pone.0289573)

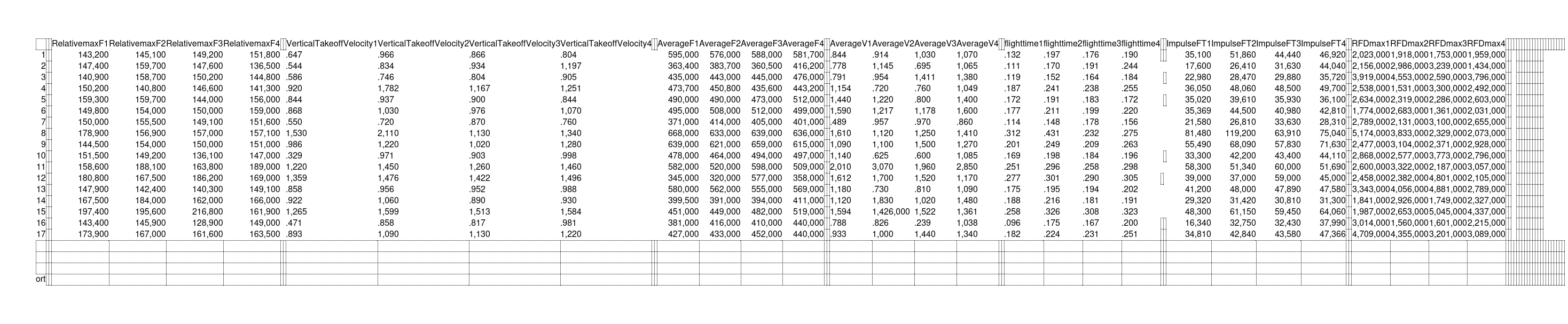

Supplement: S1 Dataset — (TIFF) [file pone.0289573.s001.tiff]
